# Supplementary material for: Role and mechanism of NCAPD3 in promoting malignant behaviors in gastric cancer
Source: Front Pharmacol. 2024 Apr 22;15:1341039. doi: 10.3389/fphar.2024.1341039 (PMC11070777; doi:10.3389/fphar.2024.1341039)
Supplement: Supplementary file 11 [file DataSheet2.ZIP › GSEA/immunologic/my_analysis.Gsea.1599462003817/GSE36888_UNTREATED_VS_IL2_TREATED_STAT5_AB_KNOCKIN_TCELL_6H_UP.html]

Details for gene set GSE36888\_UNTREATED\_VS\_IL2\_TREATED\_STAT5\_AB\_KNOCKIN\_TCELL\_6H\_UP[GSEA]

|  || Dataset | filtered\_dataset.sample\_info.cls#WT\_versus\_NCAPD3\_MUT |
| Phenotype | sample\_info.cls#WT\_versus\_NCAPD3\_MUT |
| Upregulated in class | NCAPD3\_MUT |
| GeneSet | GSE36888\_UNTREATED\_VS\_IL2\_TREATED\_STAT5\_AB\_KNOCKIN\_TCELL\_6H\_UP |
| Enrichment Score (ES) | -0.55412513 |
| Normalized Enrichment Score (NES) | -2.462653 |
| Nominal p-value | 0.0 |
| FDR q-value | 0.013282161 |
| FWER p-Value | 0.14 |
Table: GSEA Results Summary

  

Fig 1: Enrichment plot: GSE36888\_UNTREATED\_VS\_IL2\_TREATED\_STAT5\_AB\_KNOCKIN\_TCELL\_6H\_UP      
 Profile of the Running ES Score & Positions of GeneSet Members on the Rank Ordered List

  

| SYMBOL | TITLE | RANK IN GENE LIST | RANK METRIC SCORE | RUNNING ES | CORE ENRICHMENT || 1 | 10206 | TRIM13 | 161 | 0.716 | -0.0462 | No |
| 2 | 285590 | SH3PXD2B | 336 | 0.573 | -0.1155 | No |
| 3 | 81031 | SLC2A10 | 539 | 0.453 | -0.2166 | No |
| 4 | 8835 | SOCS2 | 840 | 0.275 | -0.4052 | No |
| 5 | 51278 | IER5 | 874 | -0.283 | -0.4015 | No |
| 6 | 51141 | INSIG2 | 903 | -0.328 | -0.3898 | No |
| 7 | 54541 | DDIT4 | 1133 | -0.502 | -0.5056 | Yes |
| 8 | 5359 | PLSCR1 | 1146 | -0.511 | -0.4647 | Yes |
| 9 | 4084 | MXD1 | 1167 | -0.535 | -0.4273 | Yes |
| 10 | 4301 | MLLT4 | 1237 | -0.614 | -0.4174 | Yes |
| 11 | 7057 | THBS1 | 1242 | -0.620 | -0.3602 | Yes |
| 12 | 1761 | DMRT1 | 1264 | -0.651 | -0.3124 | Yes |
| 13 | 8651 | SOCS1 | 1272 | -0.661 | -0.2534 | Yes |
| 14 | 7422 | VEGFA | 1346 | -0.788 | -0.2295 | Yes |
| 15 | 54790 | TET2 | 1364 | -0.842 | -0.1603 | Yes |
| 16 | 1577 | CYP3A5 | 1365 | -0.845 | -0.0785 | Yes |
| 17 | 7462 | LAT2 | 1399 | -1.138 | 0.0079 | Yes |
Table: GSEA details [plain text format]

  

Fig 2: GSE36888\_UNTREATED\_VS\_IL2\_TREATED\_STAT5\_AB\_KNOCKIN\_TCELL\_6H\_UP      
 Blue-Pink O' Gram in the Space of the Analyzed GeneSet

  

Fig 3: GSE36888\_UNTREATED\_VS\_IL2\_TREATED\_STAT5\_AB\_KNOCKIN\_TCELL\_6H\_UP: Random ES distribution      
 Gene set null distribution of ES for **GSE36888\_UNTREATED\_VS\_IL2\_TREATED\_STAT5\_AB\_KNOCKIN\_TCELL\_6H\_UP**
